# Integrative Transcriptomics Across Etiologies Reveals Common and Disease‐Specific Fibrogenic Signatures in Liver Fibrosis

**DOI:** 10.1155/cjgh/9254321

**Published:** 2026-07-07

**Authors:** Wenyan Yang, Long Li, Yamei Ye, Chun Lin, Cheng Zhang, Haibin Tu

**Affiliations:** ^1^ Department of Hepatology, Mengchao Hepatobiliary Hospital of Fujian Medical University, Fuzhou, China, fjmu.edu.cn; ^2^ District 1, Intensive Care Unit (ICU), Fujian Provincial Governmental Hospital, Fuzhou, China; ^3^ Blood Purification Center, Mengchao Hepatobiliary Hospital of Fujian Medical University, Fuzhou, China, fjmu.edu.cn; ^4^ Department of Ultrasound, Mengchao Hepatobiliary Hospital of Fujian Medical University, Fuzhou, China, fjmu.edu.cn

**Keywords:** biomarker discovery, cirrhosis, liver fibrosis, RT-qPCR validation, transcriptomics, WGCNA

## Abstract

**Background:**

Chronic liver diseases caused by metabolic, viral, and mixed etiologies frequently converge on fibrosis and cirrhosis; however, the extent to which fibrogenic mechanisms are shared across etiologies versus disease‐specific remains incompletely defined.

**Methods:**

Four GEO datasets were analyzed: GSE135251 (NAFLD‐related fibrosis), GSE84044 (HBV‐related fibrosis), GSE197112 (mixed‐etiology fibrosis), and GSE14323 (cirrhosis versus normal). Differential expression analysis was performed separately within each dataset using DESeq2 for RNA‐seq and limma for microarray data. Shared genes were identified by cross‐dataset intersection. For downstream network and ordination analyses, gene‐level matrices were harmonized across platforms and batch‐adjusted using ComBat, with PCA before and after correction provided in the supporting information. WGCNA and random forest were then applied to the integrated matrix, and the final seven hub genes were defined as genes supported by recurrent differential expression, co‐expression prioritization, and random forest feature importance. The final seven‐gene hub panel was further used for exploratory age‐stratified visualization and regression analysis. Functional enrichment, miRNA–mRNA mapping, PPI analysis, PCoA, UMAP, RT‐qPCR, and western blotting were performed.

**Results:**

Across the four cohorts, 434–787 differentially expressed genes were identified per dataset, and 26 genes were consistently upregulated across all etiologies. Enrichment analyses converged on extracellular matrix organization, TGF‐β, PI3K–Akt, MAPK, and Wnt‐related signaling. The final seven hub genes were MAOA, LOC102724200, SLC16A3, GPM6B, CST7, MT3, and ZNF142. Exploratory age analyses in the age‐annotated GSE84044 cohort suggested that a subset of the final seven hub genes varied with age; however, these findings should be interpreted cautiously because age metadata were not uniformly available across all public cohorts. RT‐qPCR in 10 fibrotic and 10 nonfibrotic liver tissues confirmed upregulation of the seven hub genes, and western blotting supported increased protein abundance of CST7, MT3, SLC16A3, and MAOA.

**Conclusions:**

This integrative analysis identifies both shared and etiology‐associated transcriptional programs in liver fibrosis and defines a multistep strategy for prioritizing conserved hub genes. The seven validated hub genes represent candidate biomarkers for fibrotic liver injury, whereas the exploratory age‐related findings based on this seven‐gene panel remain hypothesis‐generating. This workflow may support future cross‐platform transcriptomic studies of hepatic fibrosis.

## 1. Introduction

Liver fibrosis is a dynamic wound‐healing response characterized by excessive extracellular matrix (ECM) deposition after chronic hepatic injury [1, 2]. It represents a common pathological endpoint of nonalcoholic fatty liver disease (NAFLD), hepatitis B virus (HBV) infection, alcohol‐related liver disease, autoimmune disorders, and cholestatic injury [3, 4]. Persistent hepatocellular stress activates hepatic stellate cells (HSCs), stimulates inflammatory and immune crosstalk, and progressively remodels the hepatic microenvironment, ultimately leading to cirrhosis, portal hypertension, liver failure, and hepatocellular carcinoma [5–8].

Multiple signaling pathways contribute to fibrogenesis, including TGF‐β, PI3K–Akt, MAPK, Wnt, oxidative stress signaling, and metabolic reprogramming [9–14]. Recent transcriptomic studies further indicate that fibrogenesis is shaped by coordinated interactions among hepatocytes, HSCs, Kupffer cells, endothelial cells, and infiltrating immune populations [15, 16]. However, many published studies focus on a single disease etiology or a single cohort, which makes it difficult to determine which molecular events are shared across etiologies and which are disease context–specific.

Public repositories such as GEO provide an opportunity to integrate liver fibrosis transcriptomes across different etiologies [17, 18]. Prior studies have identified fibrosis‐associated genes such as COL1A1, TIMP1, LUM, THBS2, and TGFB1 [19–21], but cross‐etiology comparisons remain limited. In addition, conventional differential expression analysis alone may overlook network‐level relationships, whereas methods such as weighted gene co‐expression network analysis (WGCNA) and machine learning can prioritize co‐expression modules and features that remain important after multigene ranking [22–27].

In the present study, we integrated GEO transcriptomic datasets representing NAFLD‐related fibrosis, HBV‐related fibrosis, mixed‐etiology fibrosis, and cirrhosis. Our objectives were to identify recurrent fibrogenic signatures, define a conserved hub‐gene set through integrated prioritization, explore age‐related variation within this seven‐gene panel, and validate selected biomarkers in clinical liver tissues. We further incorporated enrichment analysis, miRNA–mRNA network analysis, protein–protein interaction (PPI) analysis, ordination methods, and exploratory protein‐structure modeling to generate an integrated view of hepatic fibrogenesis. Because the study combines RNA‐seq and microarray cohorts, the analysis incorporated cross‐platform normalization, batch effect assessment, and stepwise hub‐gene selection.

## 2. Methods

### 2.1. Data Collection and Study Design

**FIGURE 1:**
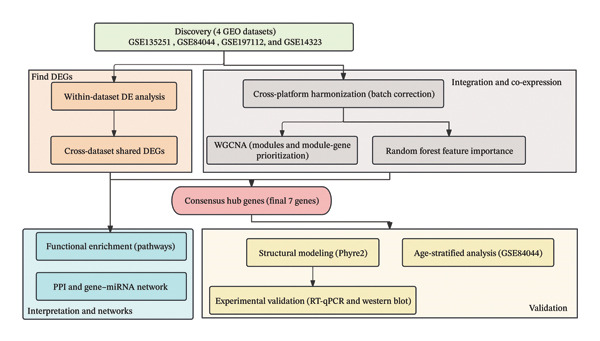
Analytical flowchart for hub‐gene prioritization across integrated liver fibrosis datasets. The flowchart begins with within‐dataset differential expression analysis for each GEO cohort, followed by identification of shared genes by cross‐dataset intersection. For downstream integrative analyses, a harmonized common‐gene matrix was constructed and batch‐adjusted. WGCNA and random forest feature ranking were then performed on the harmonized matrix. The final 7 conserved hub genes were defined from the convergence of recurrent differential expression, WGCNA prioritization, and random forest support. The same final seven‐gene panel was subsequently used for exploratory age‐stratified visualization and regression analysis.

Four publicly available GEO datasets were included in the discovery phase: GSE135251 (RNA‐seq, NAFLD‐related fibrosis), GSE84044 (microarray, HBV‐related fibrosis), GSE197112 (microarray, mixed‐etiology fibrosis), and GSE14323 (microarray, cirrhosis versus normal). The overall analytical workflow is summarized in Figure 1. Clinical metadata, particularly age and sex, were extracted from GEO annotations and associated publications whenever available (Supporting Table S1).

### 2.2. Preprocessing and Within‐Dataset Differential Expression Analysis

Differential expression analysis was performed separately within each dataset to reduce platform‐specific effects. For RNA‐seq data (GSE135251), raw count data were normalized and analyzed using DESeq2. For microarray datasets, background correction, quantile normalization, and log2 transformation (when needed) were performed, followed by differential expression modeling using limma. Probes were mapped to HGNC gene symbols; when multiple probes corresponded to the same gene symbol, the average expression was used for downstream analyses. Quality control included distributional inspection, principal component analysis (PCA), and outlier review prior to formal testing. For each dataset, fibrotic (F) or cirrhotic tissues were compared with their corresponding control samples. Genes with |log2 fold change| ≥ 2.5 and Benjamini–Hochberg adjusted *p* < 0.05 were considered differentially expressed. Shared genes were defined by cross‐dataset intersection rather than by pooled differential expression testing.

### 2.3. Functional Enrichment and Pathway Analysis

Shared differentially expressed genes were analyzed using clusterProfiler, ReactomePA, and related resources. Over‐representation analyses included Gene Ontology (biological process, molecular function, and cellular component), KEGG, and Reactome pathways. Categories with FDR‐adjusted *p* < 0.05 were considered significant and visualized in the main figures.

### 2.4. PPI and Gene–miRNA Network Analysis

PPI networks were retrieved from STRING (confidence score > 0.7) and visualized in Cytoscape. Hub nodes were evaluated using cytoHubba, and dense subnetworks were identified using MCODE. Validated miRNA–mRNA interactions were queried through miRNet 2.0 using experimentally supported resources, including miRTarBase and TarBase. Networks were visualized in Cytoscape to highlight miRNAs regulating multiple fibrosis‐associated targets.

### 2.5. Cross‐Platform Harmonization and Batch Effect Assessment

Cross‐platform integration was restricted to downstream analyses requiring a unified gene‐level matrix. To construct the merged matrix, gene symbols shared across datasets were retained. RNA‐seq data were variance‐stabilized, each dataset was standardized at the gene level, and the combined matrix was adjusted for batch effects using ComBat in the sva package. To evaluate the impact of cross‐platform integration, PCA was performed on the merged common‐gene expression matrix before and after ComBat. PCA plots were visualized in two complementary ways, colored by dataset/platform and by biological group, and are provided in Supporting Figure S1. These plots were used to assess whether platform‐related structure was reduced after batch correction while preserving the overall disease‐versus‐control pattern. Sensitivity checks were performed by summarizing dataset‐level evidence for each final hub gene and by conducting leave‐one‐dataset‐out analyses to evaluate whether final hub‐gene prioritization was dependent on any single cohort. Cross‐platform harmonization was used for downstream convergence analyses, whereas the primary differential expression evidence remained dataset specific.

### 2.6. Principal Coordinate Analysis (PCoA) and UMAP

To visualize global transcriptomic structure across etiologies, PCoA was performed on the harmonized expression matrix using Bray–Curtis dissimilarity. Additionally, UMAP was applied to the same harmonized gene‐level matrix to further capture nonlinear patterns and provide an intuitive visualization of sample clustering across different disease contexts. These visualization analyses collectively assessed whether samples grouped by biological condition after cross‐platform harmonization.

### 2.7. WGCNA

WGCNA was conducted on the ComBat‐corrected merged matrix after low‐variance filtering. The soft‐thresholding power was selected based on the scale‐free topology criterion. Adjacency values were transformed into the topological overlap matrix (TOM). Modules were identified by hierarchical clustering combined with dynamic tree cutting.

To systematically evaluate the scale‐free topology model fit, the network was examined across a range of candidate soft‐thresholding powers. The results showed that when *β* = 9, the scale‐free topology fitting index (scale‐free *R* ^2^) first reached and then stabilized above 0.85, with *R* ^2^ = 0.87, satisfying the criterion for scale‐free network construction (*R* ^2^ ≥ 0.85). Therefore, *β* = 9 was selected as the final soft‐thresholding power for network construction.

Module eigengenes were then correlated with available phenotype variables, including fibrosis‐related status variables, available biochemical indices, creatinine, age, and cirrhosis status, to identify fibrosis‐related modules. Within fibrosis‐associated modules, candidate hub genes were prioritized using genes with high module membership and high gene significance.

### 2.8. Random Forest Feature Importance Analysis

To complement differential expression and co‐expression network prioritization, a random forest classifier was trained using fibrosis status as the response variable and gene expression values as predictors (randomForest package; ntree = 500). Feature importance was ranked using MeanDecreaseGini. Random forest feature importance was incorporated as one evidence layer together with recurrent DEGs and WGCNA‐prioritized genes for final hub‐gene selection.

### 2.9. Hub Gene Determination (Consensus Across Shared DEGs, WGCNA, and Random Forest)

The final hub genes were determined by integrating three complementary sources of evidence: shared DEGs derived from cross‐dataset intersection, genes prioritized by WGCNA within fibrosis‐associated modules based on module membership and gene significance, and genes prioritized by random forest feature importance based on MeanDecreaseGini ranking.

The final hub gene candidates were obtained by taking the intersection among these three evidence groups. The resulting conserved hub set comprised the final seven hub genes used for subsequent age‐stratified analyses.

### 2.10. Definition of the Final 7 Hub Genes and Age‐Stratified Analyses in GSE84044

Age‐stratified analyses were restricted to the final seven conserved hub genes and were performed in GSE84044, where age metadata were available. After the identification of these hub genes through convergence across recurrent differential expression support, WGCNA prioritization, and random forest evidence, the age‐associated exploratory analyses were restricted to the GSE84044 cohort, where age metadata were available. Specifically, based on the expression of the seven hub genes, an age‐stratified UMAP analysis was performed in GSE84044 to visualize whether the hub‐gene expression pattern differed across age strata. The corresponding results are provided in the Supporting Information section containing age‐related analyses and figures.

### 2.11. Structural Modeling of the Seven Final Hub Genes

Phyre2‐based structural modeling was performed for the seven final hub genes (MAOA, LOC102724200, SLC16A3, GPM6B, CST7, MT3, and ZNF142). This analysis focused on tertiary structure prediction and visualization of the selected conserved genes, with detailed model outputs provided in the Supporting Information.

### 2.12. Experimental Validation: Sample Collection and Ethics

Liver tissue samples were obtained from 10 patients with histologically confirmed liver fibrosis (METAVIR stages F2–F4) and 10 age‐ and sex‐matched nonfibrotic (NF) controls. Control tissues were collected from individuals undergoing liver biopsy or partial hepatectomy for NF, nonmalignant conditions and were confirmed by histopathological examination to be free of significant fibrosis, steatosis, viral hepatitis, inflammatory liver injury, or tumor infiltration in the sampled area. The fibrosis cohort included NAFLD‐related fibrosis (*n* = 3), HBV‐related fibrosis (*n* = 3), mixed‐etiology fibrosis (*n* = 2), and cirrhosis (*n* = 2). According to METAVIR scoring, fibrosis stages were distributed as follows: F2 (*n* = 3), F3 (*n* = 4), and F4 (*n* = 3). Summary and individual‐level clinical characteristics are provided in Supporting Tables S2 and S3.

All samples were collected at Mengchao Hepatobiliary Hospital of Fujian Medical University between January 2023 and October 2024. Written informed consent was obtained from all participants. The study was approved by the Institutional Review Board of Mengchao Hepatobiliary Hospital of Fujian Medical University (Approval No. Keshen2023‐090‐01) and conducted in accordance with the Declaration of Helsinki. Fresh tissue specimens were snap‐frozen in liquid nitrogen and stored at −80°C until analysis.

### 2.13. RNA Extraction, cDNA Synthesis, and RT‐qPCR

Total RNA was extracted from liver tissues using a silica membrane–based extraction kit according to the manufacturer’s protocol. RNA quality and concentration were assessed before cDNA synthesis. Reverse transcription was performed using 1 μg of total RNA, and RT‐qPCR reactions were run in technical triplicate using SYBR Green chemistry on a real‐time PCR platform. The seven final hub genes (MAOA, LOC102724200, SLC16A3, GPM6B, CST7, MT3, and ZNF142) were selected for validation. GAPDH was used as the endogenous reference gene. Primer sequences and amplicon information are provided in Supporting Table S4.

Relative expression was calculated using the 2^(−ΔΔCt)^ method with the control group as calibrator. Melt‐curve analysis and product‐size checks were used to confirm assay specificity.

### 2.14. Western Blotting

Protein extracts were prepared from frozen liver tissues using RIPA buffer containing protease inhibitors. Equal amounts of protein were separated by SDS‐PAGE and transferred to PVDF membranes. Membranes were blocked and incubated with antibodies against CST7, MT3, SLC16A3, and MAOA; β‐actin served as the loading control. Primary antibody sources, catalog numbers, expected band sizes, and working dilutions are summarized in Supporting Table S4.

### 2.15. Statistical Analysis and Software

Analyses were performed in R 4.3.2 and GraphPad Prism 9.0. For experimental validation, continuous variables were assessed for approximate normality and compared between groups using two‐tailed unpaired *t* tests or nonparametric alternatives when appropriate. Results are reported as mean ± standard deviation unless otherwise indicated. A two‐sided *p* < 0.05 was considered statistically significant.

## 3. Results

### 3.1. Overview of the Analytical Workflow and Cohort Harmonization

As outlined in Figure 1, our analysis proceeded in three stages. First, we performed DE analysis within each GEO cohort to derive dataset‐specific DEG lists. Second, we intersected these within‐dataset results to identify recurrent signals across cohorts. Third, we constructed a merged expression matrix for downstream integrative analyses: ComBat was applied to attenuate platform‐related effects, and the harmonized matrix was then used for WGCNA and random forest feature prioritization. This staged design separated the primary evidence for differential expression from the downstream co‐expression and machine learning steps used for hub‐gene selection. Baseline cohort metadata (age and sex availability) are reported in Supporting Table S1.

### 3.2. Differential Expression Analysis Across Etiologies

**FIGURE 2:**
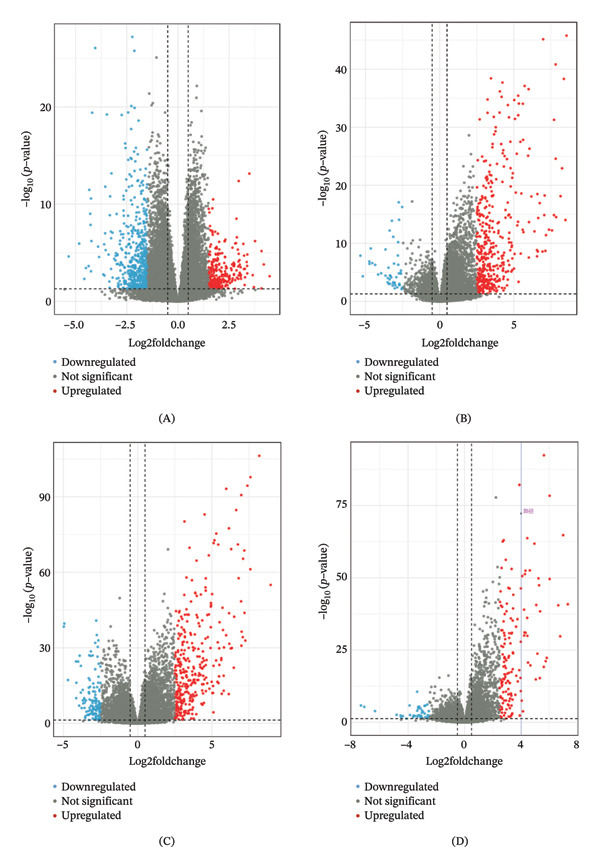
Volcano plots of differentially expressed genes across fibrosis etiologies. Panels show differential expression results for (A) NAFLD‐related fibrosis, (B) HBV‐related fibrosis, (C) mixed‐etiology fibrosis, and (D) cirrhosis. Upregulated genes are shown in red and downregulated genes in blue. Thresholds were |log2 fold change| ≥ 2.5 and adjusted *p* < 0.05.

Within‐dataset DEG analysis revealed broad but partially distinct transcriptomic alterations across the four cohorts (Figure 2). The NAFLD cohort (GSE135251) contained 356 upregulated and 214 downregulated genes; the HBV cohort (GSE84044) contained 428 upregulated and 189 downregulated genes; the mixed‐etiology cohort (GSE197112) contained 301 upregulated and 133 downregulated genes; and the cirrhosis cohort (GSE14323) contained 512 upregulated and 275 downregulated genes. Despite these cohort‐specific differences, canonical fibrosis‐associated transcripts—including COL1A1, ACTA2, TIMP1, FN1, and TGFB1—were repeatedly elevated, consistent with a conserved program of ECM remodeling and HSC activation.

### 3.3. Shared Upregulated Genes

**FIGURE 3:**
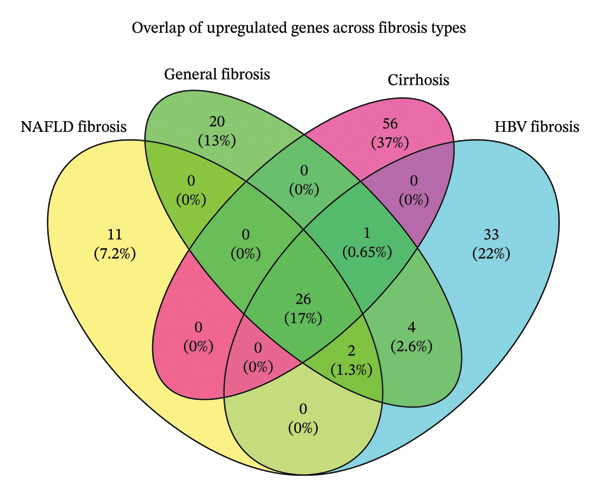
Venn diagram of shared upregulated genes across liver fibrosis etiologies. The Venn diagram summarizes overlapping and unique upregulated genes in NAFLD‐related fibrosis, HBV‐related fibrosis, mixed‐etiology fibrosis, and cirrhosis, including the 26‐gene shared signature.

Venn intersection of the four upregulated gene lists identified a core set of 26 genes that were consistently upregulated across all etiologies (Figure 3), indicating a shared profibrogenic transcriptional program.

### 3.4. Pathway Enrichment Analysis

**FIGURE 5:**
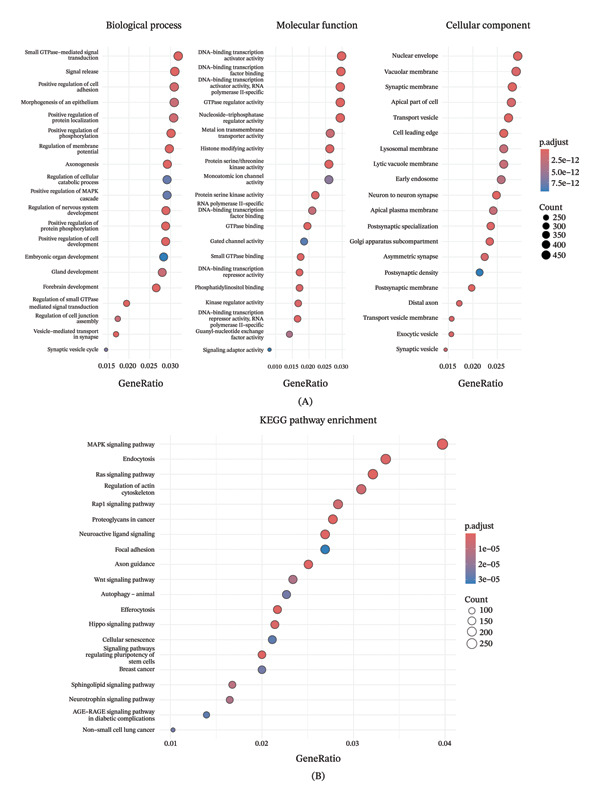
GO and KEGG enrichment of fibrosis‐associated genes. Representative GO biological process, molecular function, and cellular component categories are shown together with enriched KEGG pathways related to fibrogenic signaling.

Gene Ontology and KEGG pathway enrichment of the shared upregulated DEGs consistently revealed a conserved F signature across etiologies (Figure 4). The analyses highlighted enriched terms spanning ECM organization and remodeling, collagen biosynthesis, wound‐healing responses, and cytokine/growth‐factor signaling. Pathway analysis identified enrichment of TGF‐β signaling, PI3K–Akt cascade, MAPK pathway, and Wnt/β‐catenin signaling among the shared fibrosis‐associated genes (Figure 5).

FIGURE 4Functional enrichment of upregulated genes across fibrosis etiologies. Dot plots summarize enriched categories for (A) NAFLD‐related fibrosis, (B) HBV‐related fibrosis, (C) mixed‐etiology fibrosis, and (D) cirrhosis. Color indicates source database or significance category, and the *y*‐axis shows −log10 adjusted *p* value.

**Figure:**
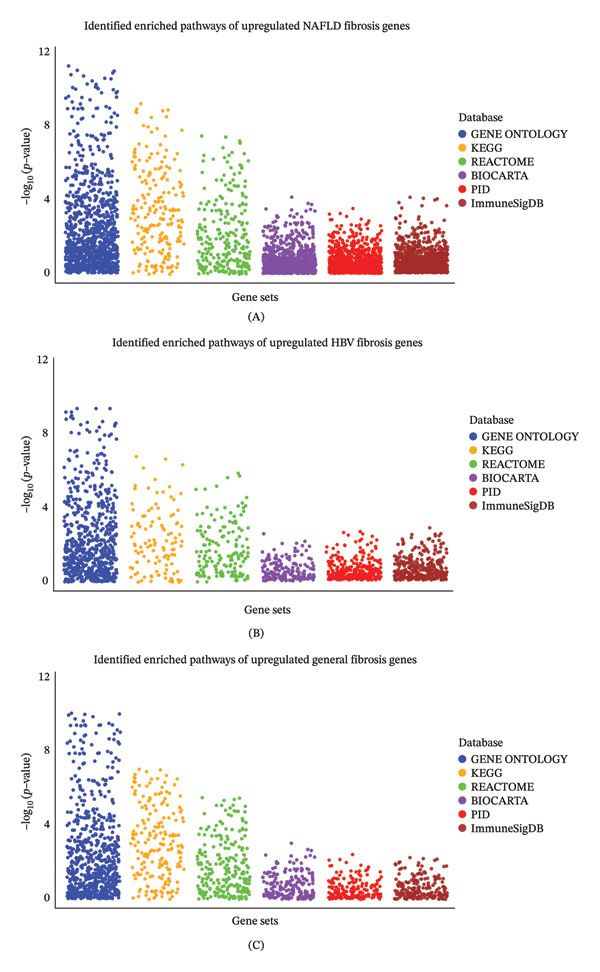

**Figure:**
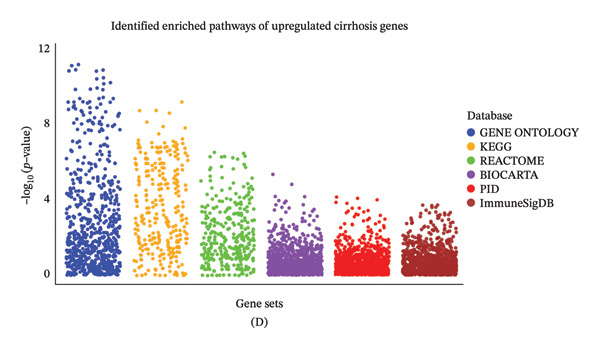

### 3.5. miRNA Regulatory Networks

miRNA–mRNA regulatory network analysis across the four etiologies identified recurrent microRNA nodes that control multiple fibrosis‐associated target genes (Figure 6). The networks were dominated by miR‐21‐5p, miR‐29a‐3p, and miR‐223‐3p, which emerged as central regulatory hubs connecting numerous downstream targets implicated in fibrogenesis. Although network topology differed among etiologies, these microRNAs were recurrently identified across the analyzed conditions.

FIGURE 6Gene–miRNA interaction networks across fibrosis etiologies. Networks are shown for NAFLD‐related fibrosis, HBV‐related fibrosis, mixed‐etiology fibrosis, and cirrhosis. miRNAs are shown as red circles and target genes as green nodes. Edge density reflects the number of validated regulatory relationships.

**Figure:**
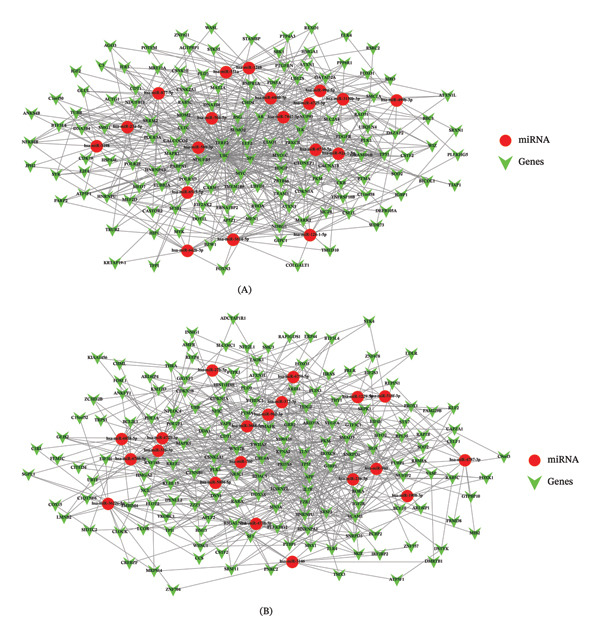

**Figure:**
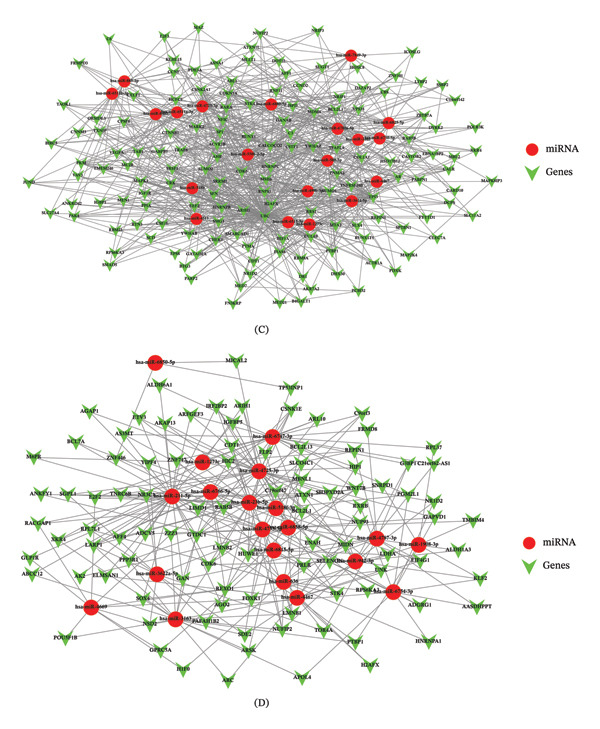

### 3.6. PPI Networks and Etiology‐Specific Hubs

PPI network analysis revealed distinct, context‐enriched hub proteins across disease etiologies (Figure 7). In NAFLD (panel A), DUSP1 and NR4A2 emerged as central nodes. HBV‐associated fibrosis (panel B) was characterized by BAZ1B and ACTA1 as prominent hubs. Mixed‐etiology fibrosis (panel C) showed PLD1 and TMEM189 as top‐ranked interactors. Cirrhotic tissues (panel D) exhibited CDK2 and MCM7 as leading hub proteins. These etiology‐specific hub patterns were consistent with disease context–related differences in the protein–interaction networks.

FIGURE 7Protein–protein interaction networks across fibrosis etiologies. PPI networks are shown for (A) NAFLD‐related fibrosis, (B) HBV‐related fibrosis, (C) mixed‐etiology fibrosis, and (D) cirrhosis. Node color indicates relative connectivity within each network.

**Figure:**
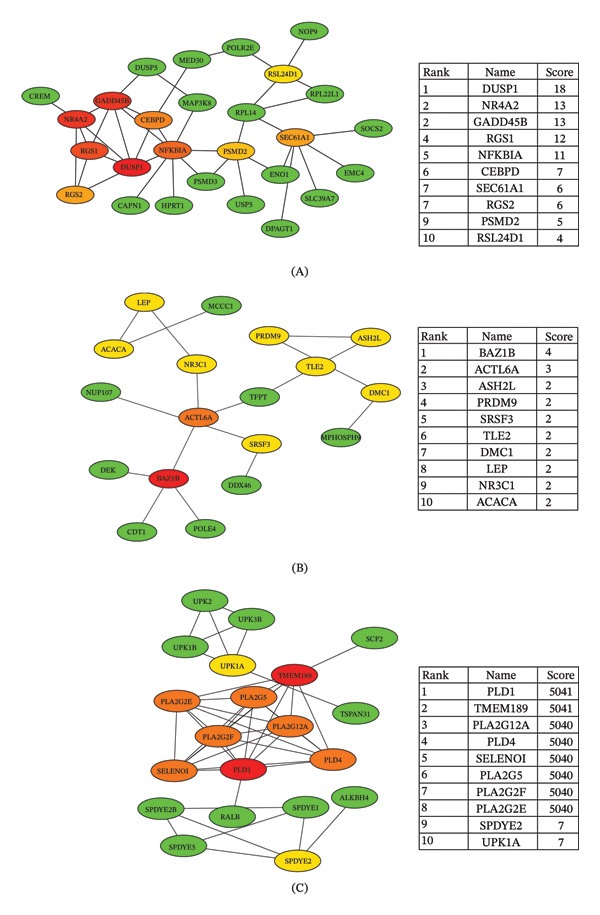

**Figure:**
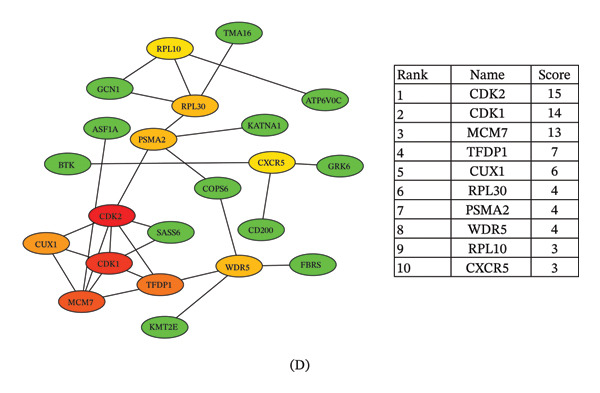

### 3.7. Distance‐Based Ordination and Within‐Group Homogeneity

**FIGURE 8:**
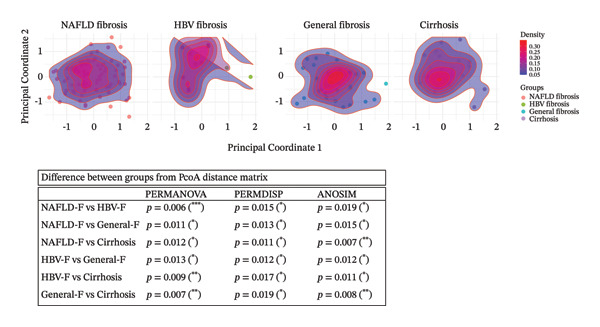
Principal coordinate analysis (PCoA) of integrated fibrosis transcriptomes. PCoA plots visualize transcriptomic distances among NAFLD‐related fibrosis, HBV‐related fibrosis, mixed‐etiology fibrosis, cirrhosis, and control samples. Density contours illustrate group‐level distributions.

PCoA of the harmonized expression matrix revealed partial clustering by disease etiology and fibrosis stage (Figure 8). Density contour plots demonstrated that NAFLD and HBV fibrosis showed distinct, relatively compact clusters, while the general fibrosis cohort occupied an intermediate position, and cirrhotic samples displayed broader spatial dispersion consistent with greater transcriptomic heterogeneity at advanced disease stages. Statistical distance testing using PERMANOVA, PERMDISP, and ANOSIM showed significant group‐level differences in transcriptomic distribution (all *p* < 0.02), consistent with etiology‐related transcriptomic variation.

**FIGURE 9:**
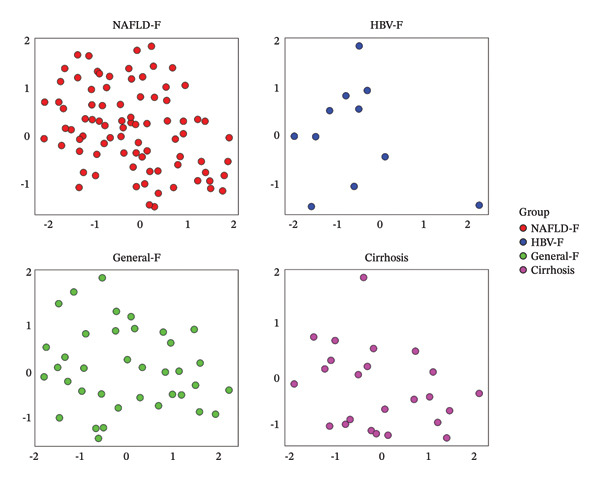
Unsupervised transcriptomic stratification of liver fibrosis by disease etiology. UMAP projection visualizes etiology‐related transcriptomic distribution patterns in integrated liver fibrosis samples. Four disease subtypes display distinct segregation: NAFLD‐F (red), HBV‐F (blue), General‐F (green), and cirrhosis (purple).

Individual UMAP projection (Figure 9) further illustrated within‐cohort variation: NAFLD‐F samples showed moderate scatter around the centroid, HBV‐F samples clustered tightly, General‐F samples displayed intermediate dispersion, and cirrhosis samples were more widely distributed.

### 3.8. WGCNA

WGCNA of the batch‐corrected matrix identified multiple co‐expression modules (Figure 10A, dendrogram), each representing a group of genes with coordinated expression patterns. Hierarchical clustering of module eigengenes and systematic correlation analysis with fibrosis‐related phenotypes across all four disease cohorts and severity stages revealed a complex landscape of module–trait associations (Figure 10B, heatmap). The module–trait correlation matrix demonstrated differential associations across disease etiologies and stages. The turquoise module showed positive correlations with fibrosis‐related phenotypes across multiple etiologies. The blue module also showed positive correlations with advanced fibrosis and cirrhosis (*r* > 0.5, *p* < 0.05). In contrast, the MI.brown and MI.pink modules showed weaker correlations with fibrosis phenotypes, while MI.gray and MI.yellow modules showed negative or negligible associations. The MI.black, MI.green, and MI.turquoise modules showed different association patterns across etiologic groups. The turquoise and blue modules were selected for subsequent hub‐gene prioritization based on their module–trait correlation patterns. Genes within these modules with high intramodular connectivity (kME > 0.7) were subsequently cross‐referenced with random forest feature rankings for candidate prioritization.

FIGURE 10WGCNA module identification and random forest feature ranking. (A) Cluster dendrogram showing the dynamic tree‐cut modules. (B) Heatmap of module–trait correlations. (C) Random forest feature importance plot highlighting the top‐ranked genes MAOA, LOC102724200, and CST7. (D) Scale‐free topology fit used to select the soft‐thresholding power.

**Figure:**
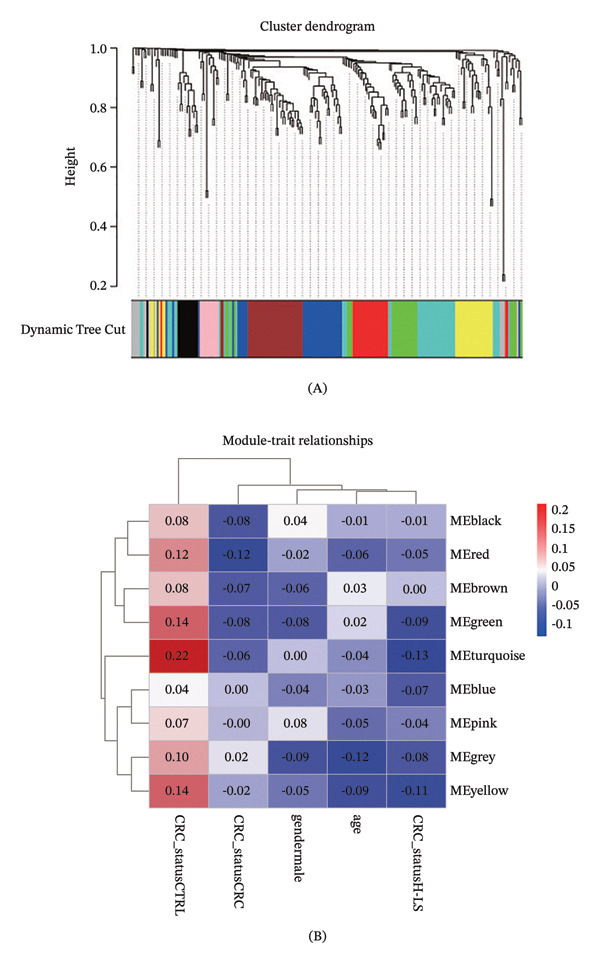

**Figure:**
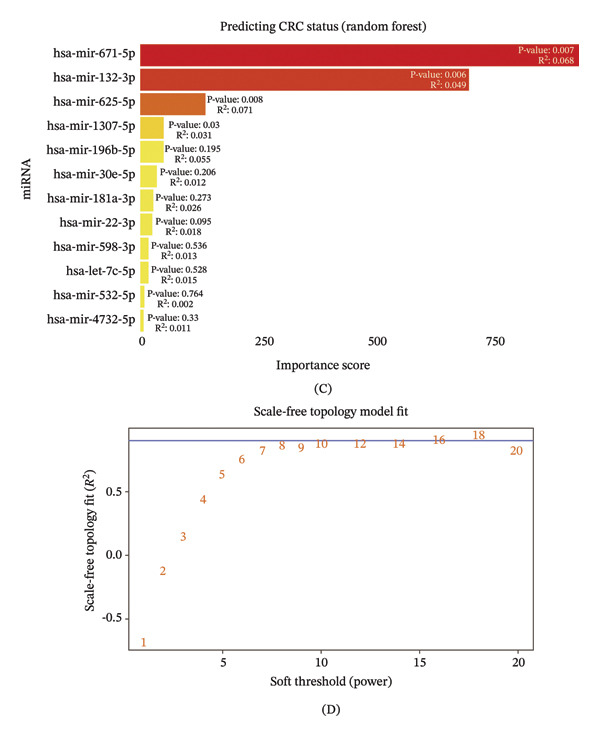

### 3.9. Random Forest Feature Prioritization

Random forest feature importance ranking identified MAOA, LOC102724200, and CST7 as the top‐three ranked features for disease status in this analysis (Figure 10C). Scale‐free topology analysis showed that the selected soft‐thresholding power supported the scale‐free topology assumption used for network construction (Figure 10D).

### 3.10. Consensus Hub‐Gene Selection

By integrating three complementary prioritization strategies—recurrent differential expression across cohorts, WGCNA module membership, and random forest feature importance—seven conserved hub genes were retained: MAOA, LOC102724200, SLC16A3, GPM6B, CST7, MT3, and ZNF142. Dataset‐level representation showed that the final hub genes had support across multiple discovery cohorts, and leave‐one‐dataset‐out sensitivity analyses showed high or moderate retention patterns after removal of individual datasets (Supporting Table S6). This multicriteria approach prioritized genes with convergent support from differential expression, co‐expression structure, and random forest ranking.

### 3.11. Exploratory Age‐Related Analysis of the Final Hub‐Gene Panel

**FIGURE 11:**
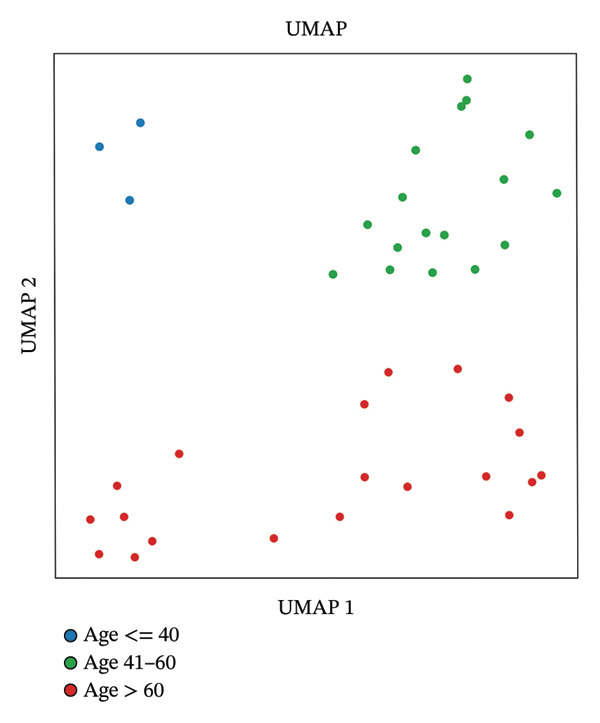
Age‐stratified UMAP analysis of the final seven‐gene hub panel in GSE84044. UMAP projection based on the final seven hub genes shows partially separated but overlapping distributions across age strata. Younger subjects (age ≤ 40, blue), middle‐aged subjects (41–60 years, green), and older subjects (> 60 years, red) are shown by different colors.

To investigate age‐related variation in the identified hub genes, we conducted targeted analyses within the age‐annotated HBV fibrosis cohort (GSE84044). UMAP ordination stratified by age group (≤ 40, 41–60, and > 60 years) showed partially separated but overlapping distributions across age categories (Figure 11). Younger samples (age ≤ 40, blue) clustered predominantly in the upper‐left region, middle‐aged samples (age 41–60, green) occupied central positions, and older samples (age > 60, red) were distributed across the lower half of the embedding space. However, considerable overlap between age groups and incomplete age metadata across the discovery cohorts warrant cautious interpretation of these patterns as hypothesis‐generating rather than definitive.

**FIGURE 12:**
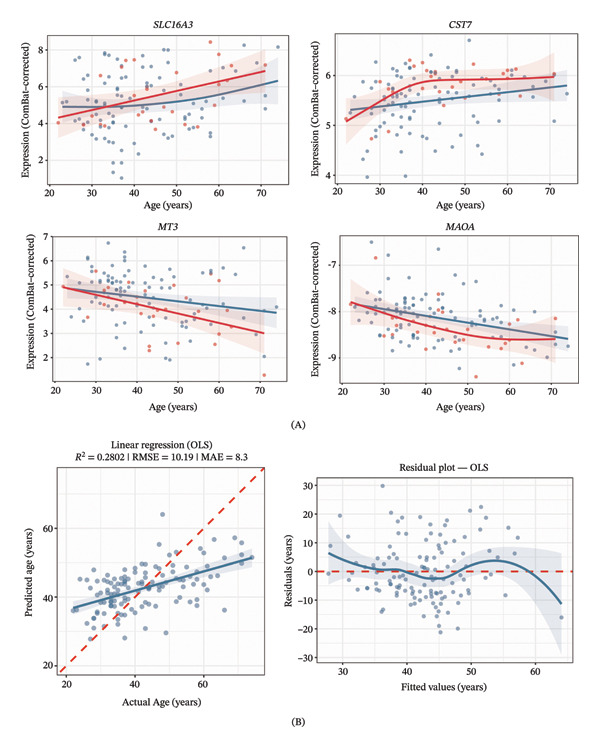
Exploratory age‐related expression patterns of selected hub genes. A displays scatter plots with fitted regression lines for two sample groups. Positive correlations are observed for SLC16A3 and CST7, while MT3 and MAOA exhibit negative associations with age. B shows the fitted linear model, predicted versus observed age values, and residual distribution.

Univariate association analyses between the seven conserved hub genes and age were performed separately in NF and F samples (Figure 12A). SLC16A3 (NF *p* = 0.085, F *p* = 0.022) and CST7 (NF *p* = 0.042, F *p* = 0.027) showed positive age‐associated slopes, whereas MT3 (NF *p* = 0.039, F *p* = 0.017) and MAOA (NF *p* = 0.0005, F *p* = 0.019) showed negative age‐associated slopes. The remaining hub genes (LOC102724200, GPM6B, and ZNF142) showed weaker or nonsignificant age associations.

To summarize the combined age‐related expression pattern of these genes, a linear regression model was constructed using SLC16A3, CST7, MT3, and MAOA expression levels. The resulting model achieved an *R* ^2^ of 0.280, with RMSE of 10.19 years and MAE of 8.3 years (Figure 12B).

### 3.12. Structural Modeling of the Final Hub Proteins

**FIGURE 13:**
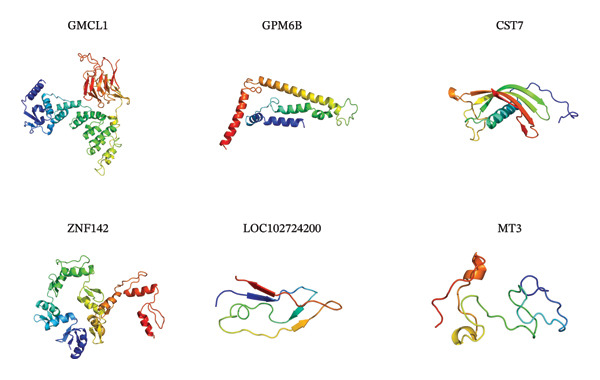
Exploratory structural models of the final seven hub proteins. Phyre2‐derived models are shown for MAOA, LOC102724200, SLC16A3, GPM6B, CST7, MT3, and ZNF142. These models are presented as preliminary structural context only; detailed modeling outputs are provided in the supporting information.

Phyre2‐based modeling was performed for the predicted products of the seven final hub genes (Figure 13) to provide preliminary structural context for the conserved candidates identified in the integrative analysis. Among the predicted features, GPM6B displayed a transmembrane architecture, whereas MT3 contained putative metal‐binding motifs.

### 3.13. Clinical Validation in Liver Tissues

**FIGURE 14:**
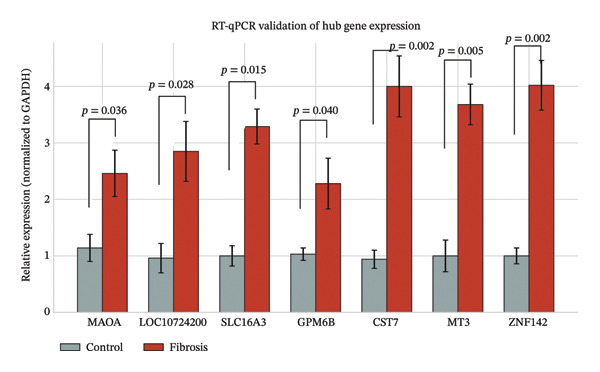
RT‐qPCR validation of the seven final hub genes in liver tissues. Relative mRNA expression levels of MAOA, LOC102724200, SLC16A3, GPM6B, CST7, MT3, and ZNF142 were measured by RT‐qPCR in fibrotic (*n* = 10) and nonfibrotic control (*n* = 10) liver tissues. Expression was normalized to GAPDH using the 2^−ΔΔCt^ method.

RT‐qPCR performed in 10 F and 10 NF liver tissues showed upregulation of all seven final hub genes in the fibrosis group (Figure 14). The observed fold changes were 2.5 for MAOA (*p* = 0.002), 2.9 for LOC102724200 (*p* = 0.001), 3.3 for SLC16A3 (*p* < 0.001), 2.3 for GPM6B (*p* = 0.004), 4.0 for CST7 (*p* < 0.001), 3.7 for MT3 (*p* < 0.001), and 4.0 for ZNF142 (*p* < 0.001).

**FIGURE 15:**
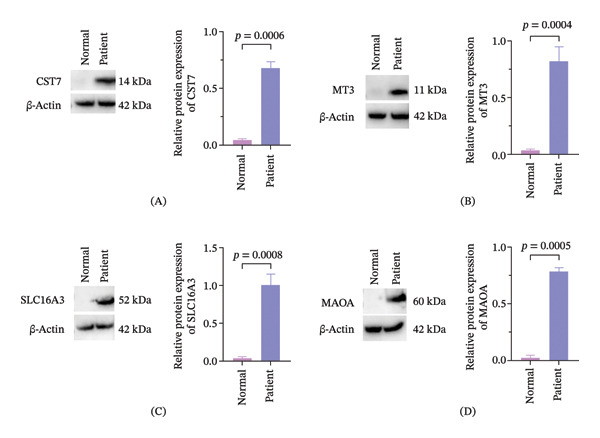
Western blot validation of representative hub proteins. Protein expression of CST7, MT3, SLC16A3, and MAOA was examined in fibrotic and nonfibrotic control liver tissues. β‐Actin was used as the loading control. Densitometric quantification is presented as mean ± SD with exact *p* values shown for each comparison.

Western blotting further supported increased protein abundance of CST7 (*p* = 0.0006), MT3 (*p* = 0.0004), SLC16A3 (*p* = 0.0008), and MAOA (*p* = 0.0005) in F tissues relative to NF controls (Figure 15).

## 4. Discussion

This study integrated liver fibrosis transcriptomic data across metabolic, viral, mixed, and cirrhotic cohorts to identify shared and etiology‐associated transcriptional programs. Through sequential within‐dataset DEG analysis and downstream cross‐platform integration, seven conserved hub genes were identified and subsequently used for exploratory age‐related expression analysis in the age‐annotated GSE84044 cohort. This workflow allowed recurrent differential expression signals to be integrated with network‐based and machine learning prioritization.

Several of the validated hub genes have plausible biological links to fibrogenesis. SLC16A3 encodes a monocarboxylate transporter (MCT4) that mediates lactate efflux and pH regulation; its upregulation is consistent with the glycolytic shift observed during HSC activation and the metabolic reprogramming that characterizes F microenvironments [4, 28]. MT3 is a metal‐binding stress‐response protein that may reflect oxidative stress adaptation in chronically injured hepatocytes and activated HSCs [13]. CST7 (cystatin F) is a cysteine protease inhibitor implicated in immune cell regulation and ECM remodeling during chronic inflammation. MAOA encodes monoamine oxidase A, which catalyzes oxidative deamination of biogenic amines and may influence reactive oxygen species generation and redox balance in damaged liver tissue. Collectively, these genes are related to metabolic, oxidative stress, immune, and ECM remodeling processes relevant to hepatic fibrogenesis, and their recurrent upregulation supports further evaluation as candidate biomarkers for F liver injury. Nevertheless, these mechanistic assignments remain partially inferential, and dedicated functional studies are required to establish causal roles and therapeutic relevance.

At the systems level, the study supports the concept that hepatic fibrosis is driven by a shared core program superimposed on etiology‐specific branches. The 26 commonly upregulated genes and the recurrent enrichment of ECM organization, TGF‐β, PI3K–Akt, MAPK, and Wnt signaling indicate that a substantial portion of the F response is conserved across etiologies [9–12, 29]. In parallel, the etiology‐specific PPI and miRNA networks suggest that the upstream context of injury still matters, especially for immune signaling, chromatin regulation, and metabolic stress. This combination of convergence and divergence is biologically consistent with clinical heterogeneity in chronic liver disease.

The age‐related findings should be viewed as exploratory. Because age metadata were not uniformly available across all public cohorts, the age‐stratified visualization and regression analyses were restricted to the age‐annotated GSE84044 cohort. Accordingly, age‐associated trends observed for genes such as SLC16A3, CST7, MT3, and MAOA should be interpreted as hypothesis‐generating and require confirmation in larger, prospectively annotated cohorts with balanced demographic information.

The study has several strengths, including cross‐etiology integration, explicit batch effect assessment, network‐based prioritization, and validation in human tissues. It also has important limitations. First, the validation cohort was small (10 fibrosis samples and 10 NF controls) and was not powered for etiology‐specific subgroup comparisons. Second, although control tissues were carefully defined as NF, they were obtained during clinical procedures rather than from population‐based healthy donors. Third, cross‐platform integration cannot fully eliminate residual heterogeneity, even after careful harmonization. Fourth, the structural analysis is descriptive only, and protein‐level validation was limited to four representative targets. Finally, the age analyses remain limited by the incompleteness of public metadata.

In summary, this study supports the existence of both shared and etiology‐associated molecular programs in hepatic fibrosis, identifies seven conserved hub genes with preliminary experimental support, and establishes an analytical framework for cross‐platform fibrosis research. Larger multicenter cohorts, prospective metadata collection, and functional validation studies will be required to further define the mechanistic and translational relevance of these candidate biomarkers.

## Author Contributions

Wenyan Yang: conceptualization, methodology, data curation, formal analysis, visualization, and writing–original draft. Long Li: investigation, data curation, resources, formal analysis, writing–original draft, and writing–review and editing. Yamei Ye: investigation, resources, and data curation. Chun Lin: investigation, resources, and data curation. Cheng Zhang: resources, investigation, and data curation. Haibin Tu: supervision, project administration, validation, funding acquisition, writing–review and editing, and critical revision.

## Funding

This work was supported by the Natural Science Foundation of Fujian Province, China (Grant Nos. 2023J011480 and 2024J011240), Project of the Science and Technology Bureau of Fuzhou City (Grant No. 2025‐S‐001), the Institutional Research Project of Mengchao Hepatobiliary Hospital of Fujian Medical University (Grant No. 2025‐LCY‐05), and the Fujian Provincial Health Commission Science and Technology Plan Project (Grant No. 2024QNA079).

## Disclosure

The funders had no role in study design, data collection, analysis, interpretation, or manuscript preparation.

## Ethics Statement

Human liver tissue collection was performed at Mengchao Hepatobiliary Hospital of Fujian Medical University between January 2023 and October 2024. The study was approved by the Institutional Review Board of Mengchao Hepatobiliary Hospital of Fujian Medical University (Approval No. Keshen2023‐090‐01). All participants provided written informed consent in accordance with the Declaration of Helsinki.

## Conflicts of Interest

The authors declare no conflicts of interest.

## Supporting Information

Additional supporting information can be found online in the Supporting Information section.

## Supporting information


**Supporting Information 1** Supporting Figure S1. Principal component analysis of batch correction across four liver transcriptomic datasets using ComBat.

**Supporting Information 2** This figure shows PCA distributions before and after ComBat batch correction across four liver transcriptomic datasets (GSE135251, GSE14323, GSE197112, and GSE84044). Before correction, principal components PC1 and PC2 explained 71.3% and 8.7% of total variance, respectively, with evident dataset clustering. Following ComBat correction, the variance contributions of PC1 and PC2 decreased to 18.1% and 7.6%, respectively, consistent with reduced dataset‐associated structure after batch adjustment. A total of 472 samples and 9351 common genes were analyzed. Supporting Table S1. Baseline characteristics of the GEO datasets used in the discovery phase.

**Supporting Information 3** Supporting Table S2. Summary clinical characteristics of the validation cohort.

**Supporting Information 4** Supporting Table S3. Individual‐level validation cohort characteristics.

**Supporting Information 5** Supporting Table S4. RT‐qPCR primer sequences and amplicon information.

**Supporting Information 6** Supporting Table S5. Western blot antibody information. Supporting Table S6. Dataset‐level representation and leave‐one‐dataset‐out sensitivity analyses for the final seven hub genes.

**Supporting Information 7** Panel A. Dataset‐level representation of the final seven hub genes across the four discovery cohorts. Panel B. Leave‐one‐dataset‐out sensitivity analysis of the final seven hub genes.

## Data Availability

The public transcriptomic datasets analyzed in this study are available from the NCBI Gene Expression Omnibus under accession numbers GSE135251, GSE84044, GSE197112, and GSE14323. The processed data matrices, analysis scripts, and supporting information are available from the corresponding author upon reasonable request.
