# Supplementary material for: Integrative Transcriptomics Across Etiologies Reveals Common and Disease‐Specific Fibrogenic Signatures in Liver Fibrosis
Source: Can J Gastroenterol Hepatol. 2026 Jul 7;2026:9254321. doi: 10.1155/cjgh/9254321 (PMC13341641; doi:10.1155/cjgh/9254321)
Supplement: Supplementary file 1 — Supporting Information 1 Supporting Figure S1. Principal component analysis of batch correction across four liver transcriptomic datasets using ComBat. [file CJGH-2026-9254321-s004.pdf]

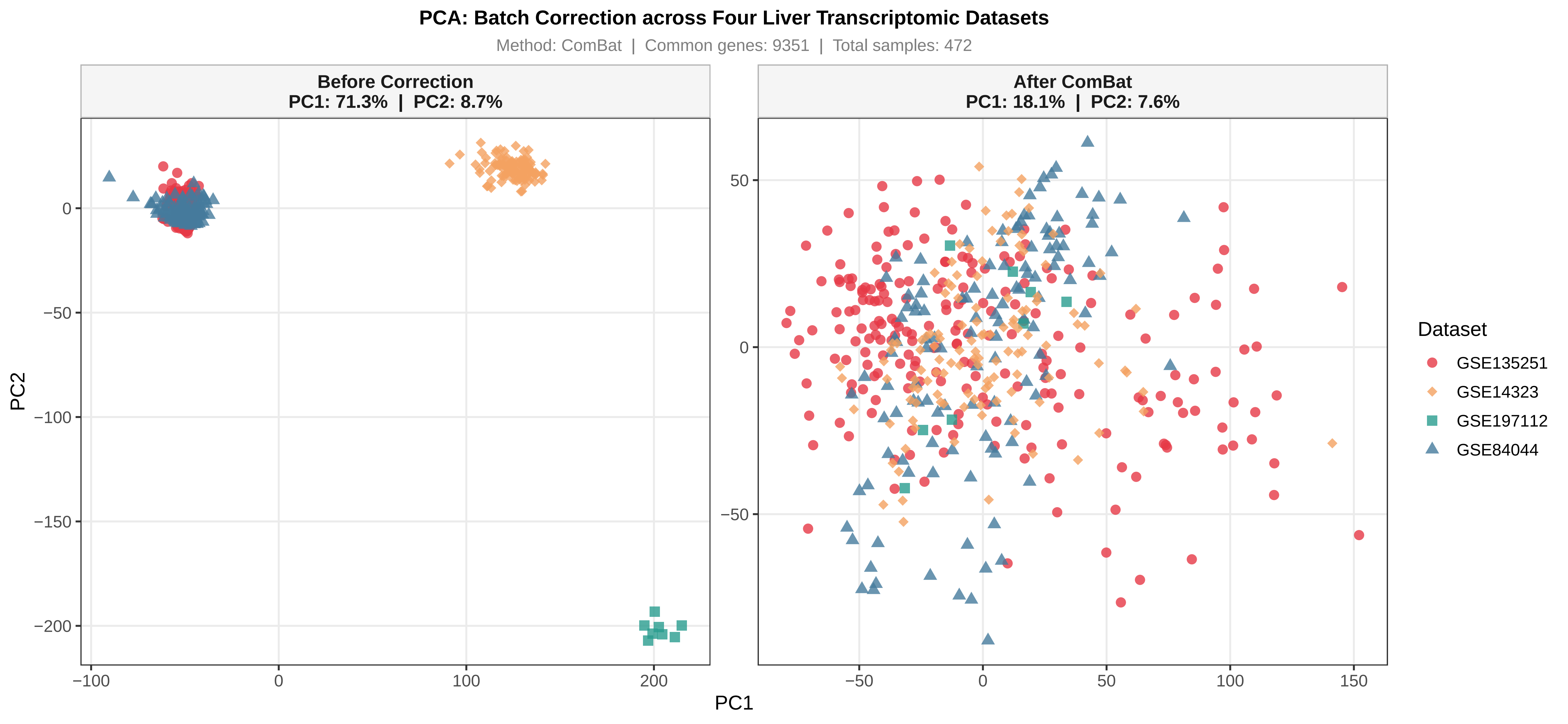
PCA: Batch Correction across Four Liver Transcriptomic Datasets
Method: ComBat | Common genes: 9351 | Total samples: 472
Before Correction
PC1: 71.3% | PC2: 8.7%
After ComBat
PC1: 18.1% | PC2: 7.6%
PC2
PC1
0
−50
−100
−150
−200
−100
0
100
200
50
0
−50
−50
0
50
100
150
Dataset
GSE135251
GSE14323
GSE197112
GSE84044
